# Antimicrobial Susceptibility of *Glaesserella parasuis* to Macrolides and Characterization of *erm*(T)-Carrying Mobile Elements on Chromosome

**DOI:** 10.3390/ani15020164

**Published:** 2025-01-10

**Authors:** Peng Zhang, Changmin Li, Shuna Shang, Ting Huang, Junqi Liu, Qianwen Ge, Xiaoping Liao, Liangxing Fang, Yang Yu

**Affiliations:** 1 National Risk Assessment Laboratory for Antimicrobial Resistance of Animal Original Bacteria, South China Agricultural University, Guangzhou 510642, China; cxyzzhangpeng@163.com (P.Z.); 15738737393@163.com (C.L.); 15626191906@163.com (S.S.); 18568330723@163.com (J.L.); geqianwen_scau@163.com (Q.G.); xpliao@scau.edu.cn (X.L.); fanglx@scau.edu.cn (L.F.); 2 Veterinary Medicine, College of Animal Science and Technology, Zhongkai University of Agriculture and Engineering, Guangzhou 510225, China; htjinan@163.com; 3 Guangdong Provincial Key Laboratory of Veterinary Pharmaceutics Development and Safety Evaluation, South China Agricultural University, Guangzhou 510642, China; 4 National Reference Laboratory of Veterinary Drug Residues, Veterinary Pharmacology Department, College of Veterinary Medicine, South China Agricultural University, Guangzhou 510642, China

**Keywords:** *Glaesserella parasuis*, macrolide resistance, *erm*(T), integrative and conjugative element

## Abstract

*Glaesserella parasuis* is the etiological agent of Glässer’s disease, which causes high morbidity and mortality in pigs worldwide. Macrolide resistance poses an urgent threat to their treatment, as macrolides are widely used for preventing and treating *G. parasuis* infections. Here, we determined the susceptibilities to five macrolides and characterized the genetic markers of macrolide resistance. The antimicrobial susceptibility of 117 *G. parasuis* isolates to erythromycin, tulathromycin, gamithromycin, tylosin, and tilmicosin was evaluated using broth microdilution method. Erythromycin-resistant isolates were sequenced using whole-genome sequencing. Further analysis of these sequences revealed the genetic basis of macrolide resistance in *G. parasuis*. Our results show that most *G. parasuis* isolates remained susceptible to the macrolide drugs. For commonly used agents (e.g., tylosin and tilmicosin), elevated minimum inhibitory concentrations (MICs) were observed, whereas for the newer macrolides (e.g., tulathromycin and gamithromycin), the MICs remained almost unchanged. The macrolide resistance gene *erm*(T) and the A2059G mutation in 23S rRNA were detected in the current study. To the best of our knowledge, integrative and conjugative element (ICE)-borne *erm*(T) in *G. parasuis* is reported for the first time in this study. Taken together, these results provide insights into the susceptibility of *G. parasuis* to macrolides. The presence of *erm*(T) on ICEs may facilitate its transfer, reducing the effectiveness of macrolide treatment.

## Simple Summary

*Glaesserella parasuis* is an early colonizer of the upper respiratory tract of pigs and causes high morbidity and mortality. Although macrolides have gained wide clinical application for the treatment of *G. parasuis* infections, the susceptibility profiles of different macrolide drugs have not been extensively compared. We evaluated the comparative sensitivity of 14-, 15-, and 16-membered macrolides against *G. parasuis*, and investigated the associated resistance mechanisms. Comprehensive data on the macrolide susceptibility of *G. parasuis* were obtained. In addition, the presence of integrative and conjugative element (ICE)-borne *erm*(T) was also a novel observation. Our results provide valuable insights into macrolide resistance in *G. parasuis* and may guide empirical treatment recommendations.

## 1. Introduction

*Glaesserella parasuis* is a member of the family *Pasteurellaceae* and an early colonizer of the upper respiratory tract of healthy pigs, comprising virulent and non-virulent strains [1,2]. Under certain circumstances, the virulent strains can cause Glässer’s disease, characterized by polyarthritis, fibrinous polyserositis, and meningitis, resulting in economic losses and reduced pig welfare [3]. Macrolides are widely used to control such infections. However, misuse of these agents drives the emergence of antimicrobial resistance (AMR) and selects for resistant groups, making the treatment of pigs ineffective and leading to more severe infections [4].

To address the threat posed by AMR, it is important to conduct complete susceptibility tests for macrolides and detect the genetic basis of resistance to the agents [5]. Several previous studies demonstrated the susceptibility of *G. parasuis* to certain macrolides, such as erythromycin, tylosin, tilmicosin, and tulathromycin, whereas few have focused on gamithromycin, an azalide macrolide drug that was recently approved for the treatment and control of respiratory diseases [6,7,8,9]. *G. parasuis* isolates are sensitive to macrolides, and only a few resistant strains harbor macrolide resistance genes or mutations [10,11].

The macrolide resistance known to occur in *Pasteurellaceae* is mediated by rRNA methylases, mutations in ribosomal proteins and 23S rRNA, and active efflux or inactivation by phosphotransferases [12]. More recently, *estT* conferring resistance only to 16-membered macrolides was identified on a plasmid in *Sphingobacterium faecium* [13]. The *erm*(T) gene has been identified preferentially in Gram-positive genera such as *Lactobacillus*, *Streptococcus*, *Staphylococcus*, *Enterococcus*, and *Erysipelothrix*, as well as in Gram-negative genera such as *Glaesserella*, *Mannheimia*, and *Acinetobacter* [14]. In most cases, *erm*(T) is detected in various plasmids. Recently, integrative and conjugative element (ICE)-borne *erm*(T) was identified in some genera, such as *Streptococcus suis* and *Mannheimia haemolytica* [14,15]. In isolates of *G. parasuis*, plasmid-borne *erm*(T) and the A2059G mutation in 23S rRNA were reported to be responsible for macrolide resistance [10,16]. Notably, several recent studies showed that AMR genes were frequently associated with ICEs in *G. parasuis*, and were capable of being transferred between different bacterial species [17].

In the present study, we determined the susceptibility of 117 clinical *G. parasuis* isolates to 14-, 15-, and 16-membered macrolides and characterized the genetic environment of ICE-borne *erm*(T). Our objective was to comprehensively understand macrolide resistance phenotypes and characterize the genetic environment of *erm*(T). This study provides valuable insights into macrolide resistance in *G. parasuis* and may guide empirical treatment recommendations.

## 2. Materials and Methods

### 2.1. Strains and Culture Conditions

All 117 *G. parasuis* isolates were supplied by the National Reference Laboratory of Veterinary Drug Residues (Guangzhou, China) and were isolated from pigs with polyserositis, pneumonia, or septicemia in South China between 2010 and 2017. *G. parasuis* isolates were cultured on tryptic soy agar supplemented with 10 μg/mL nicotinamide adenine dinucleotide and 5% (*v*/*v*) fetal calf serum. The isolates were identified using 16S diagnostic polymerase chain reaction [18].

### 2.2. Antimicrobial Susceptibility Testing

The antimicrobial susceptibility of the 117 isolates was determined using the broth microdilution method as described previously [19]. Briefly, the inocula were prepared from 24 h supplemented tryptic soy agar by adjusting to a 0.5 McFarland standard and further diluted to 1:200 in supplemented cation-adjusted Mueller–Hinton broth containing 25 μg/mL nicotinamide adenine dinucleotide and 1% (*v*/*v*) sterile filtered heat-inactivated chicken serum. The adjusted inoculum (50 μL) was added to each well of a microtiter plate. Microtiter plates were evaluated visually after 24 h of incubation in an ambient-air incubator.

Five macrolides were used for susceptibility testing with the following dilution ranges: erythromycin (14-membered macrolide), 0.25–128 μg/mL; tulathromycin (15-membered macrolide), 0.25–128 μg/mL; gamithromycin (15-membered macrolide), 0.25–128 μg/mL; tylosin (16-membered macrolide), 0.25–128 μg/mL; and tilmicosin (16-membered macrolide), 0.25–128 μg/mL. The quality control strains were *Actinobacillus pleuropneumoniae* ATCC 27090 and *Staphylococcus aureus* ATCC 29213. As no species-specific breakpoints were available [20], the 50% and 90% minimum inhibitory concentrations (MIC_50_ and MIC_90_, respectively) were determined for each drug. Isolates with an erythromycin MIC of >4 μg/mL were classified as “resistant” and subsequently evaluated using whole-genome sequencing.

### 2.3. Genomic DNA Extraction and Whole-Genome Sequencing

Five erythromycin-resistant isolates were cultured on supplemented tryptic soy agar at 37 °C for 18–24 h, and colonies were scraped from the plates for genomic DNA extraction. Genomic DNA was extracted using a TIANamp Bacteria DNA Kit (Tiangen, Beijing, China) following the manufacturer’s instructions. The extracted DNA was used for whole-genome sequencing with 250 bp reads on a MiSeq platform (Illumina, San Diego, CA, USA). The Trimmomatic output was used for de novo assembly using SPAdes v3.13.0 [21]. The *G. parasuis* strain H68tg with a contig that was too short (3824 bp) containing *erm*(T) was also selected for long-read sequencing on the Oxford Nanopore Technologies GridION Platform (Nanopore, Oxford, UK). Both short (San Diego, CA, USA) and long (Oxford, UK) reads were used for hybrid de novo whole-genome assembly using Unicycler v.0.5.0 with default settings [22].

### 2.4. Identification of Macrolide Resistance Genes and Mutations

Whole-genome sequences were screened for known macrolide resistance genes using ResFinder v4.6.0 http://genepi.food.dtu.dk/resfinder (accessed on 10 October 2024) with default settings, a threshold for %ID of 90%, and a minimum length of 60%. The 16-membered macrolide resistance gene *estT* was not available in the resistance gene database; therefore, we downloaded its reference sequence for *S. faecium* strain WB1 plasmid pWB1 (CP094932.1) from the NCBI database. Chromosomal mutations associated with macrolide resistance were determined in L4 and L22 ribosomal proteins, as well as in 23S rRNA, using online BLAST software v2.16.0 https://blast.ncbi.nlm.nih.gov/Blast.cgi (accessed on 10 October 2024). *G. parasuis* Nagasaki (CP018034.1) was used as the reference strain. *Escherichia coli* K-12 substrain MG1655 was used for the numbering of nucleotides.

### 2.5. Mobile Element Analysis

*erm*(T)-containing genome sequences were submitted to ICEberg 3.0 to identify putative ICEs [23]. Manual curation and comparison of putative ICEs with ICE*Hpa1* were performed using SnapGene v.6.0.2 and Easyfig v.2.2.5.

## 3. Results and Discussion

### 3.1. Antimicrobial Susceptibilities of Five Macrolides

As there were no species-specific breakpoints available for *G. parasuis*, the strains could not be defined as resistant or susceptible. The distribution of the MICs as well as the MIC_50_ or MIC_90_ values was used for comparison (Table 1).

For erythromycin, 5 of the 117 isolates showed higher MICs than the others. The MIC_50_ and MIC_90_ values of erythromycin were 1 and 4 μg/mL, respectively, which were slightly higher than those reported by Zhou et al. (0.5 and 2 μg/mL, respectively, for strains isolated during 2007–2008), but lower than those reported by Zhang et al. (2 and 16 μg/mL, respectively, for strains isolated during 2016–2017) [7,8]. Elevated MICs were also observed for the older macrolides, tylosin and tilmicosin, with MIC_90_ values of 32 and 8 μg/mL [7,8], possibly because of the overuse of these two drugs in China.

For the newer macrolides (e.g., tulathromycin and gamithromycin), particularly gamithromycin, most isolates had lower MICs than those of extensively used agents (e.g., tylosin and tilmicosin). A study was previously conducted in Australia to investigat *G. parasuis* isolates collected between 2002 and 2013 for their susceptibility to tulathromycin [24]. The study revealed that Australian isolates of *G. parasuis* also had low MIC_50_ and MIC_90_ values (1 and 8 μg/mL) for tulathromycin. A study in China in which *G. parasuis* isolates were tested for their susceptibility to gamithromycin showed that the MIC_90_ value was extremely low (1 μg/mL), which was in accordance with our results [25].

### 3.2. Macrolide Resistance Genes and Mutations

A total of five *G. parasuis* isolates were “resistant” to erythromycin and were sequenced (Table 2). No acquired macrolide resistance genes or mutations were detected in strain 20. One strain, H62, exhibited high MICs for all the macrolides included in this study. Analysis of the whole-genome sequence of this isolate revealed the presence of the A2059G mutation in 23S rRNA. The mutation A2059G in *G. parasuis* was reported for the first time by Dayao et al. [16]. Since then, this mutation has been reported less frequently in *G. parasuis*. For the L4 and L22 proteins, we found mutations in isolates with both low and high macrolide MICs (data shown in another study, unpublished).

The macrolide resistance gene *erm*(T) was identified in three of the five sequenced isolates. All *erm*(T)-containing strains (59, H44, and H68tg) showed elevated MICs for the macrolides tested. Strain H44 had higher MICs for tulathromycin and gamithromycin, but similar MICs for other macrolides, which may not be explained solely by the presence of *erm*(T). Since the first report of plasmid-borne *erm*(T) in *G. parasuis*, this gene has rarely been reported in this species in recent years [10]. In *S. suis*, *erm*(T) also has a low detection rate, possibly because of the fitness cost of *erm*(T)-carrying plasmids or ICEs [14].

### 3.3. Characterization of erm(T)-Carrying ICEs

**Figure 1:**
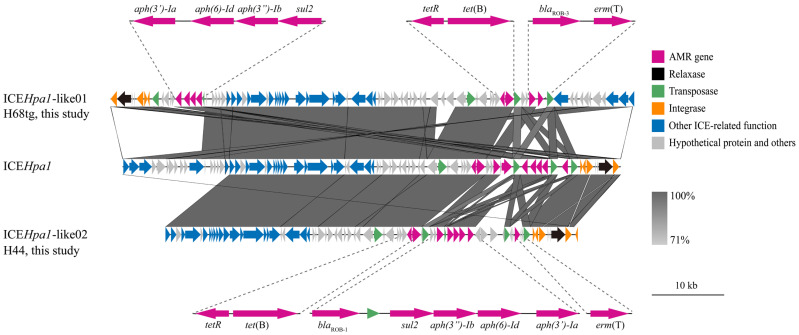
Putative ICEs identified in this study: ICE*Hpa1*-like01 and ICE*Hpa1*-like02. AMR gene, relaxase, transposase, integrase, ICE-related function sequences, and hypothetical proteins are indicated by colors according to the legend. Scale bar represents 10 kb pairs. AMR, antimicrobial resistance; ICEs, integrative and conjugative elements.

One of the three *erm*(T)-carrying isolates (strain 59) was previously reported [10]. The other two isolates were completely sequenced, and *erm*(T) was identified in sufficiently long contigs (2,367,942 bp for H68tg, 231,690 bp for H44) on the chromosome. ICEfinder identified putative ICEs in both the H44 and H68tg genome sequences. Both contained the macrolide resistance gene *erm*(T) and shared common backbones with ICE*Hpa1* (CP054198.1). Compared with ICE*Hpa1*, they had different cargo genes and were designated as ICE*Hpa1*-like01 (H68tg) and ICE*Hpa1*-like02 (H44) (Figure 1). ICE*Hpa1* was first detected in the *G. parasuis* strain YHP170504 [26]. Diverse ICEs from the ICE*Hpa1* family are prevalent in *G. parasuis* [17].

ICE*Hpa1*-like01 was 72,249 bp in size and contained seven antimicrobial resistance genes, which conferred resistance to macrolides (*erm*(T)), β-lactams (*bla*_ROB-3_), tetracyclines (*tet*(B)), sulfonamides (*sul2*), and aminoglycosides (*aph(3″)-Ib*, *aph(6)-Id*, and *aph(3′)-Ia*). Compared with ICE*Hpa1*, ICE*Hpa1*-like01 carried *erm*(T) but not *aac(6′)-aph(2″)*. *erm*(T) and *bla*_ROB-3_ are flanked by two IS*Apl1* elements. IS*Apl1* was originally identified in *A. pleuropneumoniae* from pigs [27] and has been frequently observed to flank AMR genes in the ICE*Hpa1* family in *G. parasuis*, as well as in other *Pasteurellaceae* of animal origin [17,28,29]. Therefore, the IS*Apl1*-*bla*_ROB-3_-*erm*(T)-IS*Apl1* structure may facilitate the transfer of *erm*(T) between different *Pasteurellaceae* species.

ICE*Hpa1*-like02 is 57,037 bp in size and carries AMR genes similar to ICE*Hpa1*-like01. However, the fragment IS4-like-*tetR*-*tet*(B)-IS*Apl1-bla*_ROB-1_*-*ΔIS*Apl1-sul2-aph(3″)-Ib-aph(6)-Id-aph(3′)-Ia*-ΔIS*Apl1-erm*(T)-IS*Apl1* differed from ICE*Hpa1*-like01, as well as those reported previously [26,30,31]. ICE*Hpa1*-like02 had two IS*Apl1* copies flanking several genes, conferring resistance to β-lactams, sulfonamides, aminoglycosides, and macrolides. Moreover, four copies of IS*Apl1* were identified in this structure, which may form various circular intermediates and transfer between bacterial species through a “copy-out-paste-in” mechanism [29].

### 3.4. Limitations

We determined the mechanisms underlying macrolide resistance and characterized the *erm*(T)-containing ICEs. However, we did not determine the transferability of these two putative ICEs. Further studies are needed to evaluate the transferability, fitness costs, and other molecular traits.

## 4. Conclusions

Most *G. parasuis* isolates remained susceptible to macrolide drugs. For commonly used agents (e.g., tylosin and tilmicosin), elevated MICs were observed, whereas for the newer macrolides (e.g., tulathromycin and gamithromycin), the MICs remained almost unchanged. The macrolide resistance gene *erm*(T) and the A2059G mutation in 23S rRNA were detected in the current study. To the best of our knowledge, ICE-borne *erm*(T) in *G. parasuis* is reported for the first time in this study. Taken together, these results provide insights into the susceptibility of *G. parasuis* to macrolides. The presence of *erm*(T) on ICEs may facilitate its transfer which will reduce the effectiveness of macrolide treatment.

## Figures and Tables

**Table 1:** MIC distribution of 117 *Glaesseralla parasuis* isolates for macrolides.

Antibiotics	No. of Isolates with MICs (μg/mL)											
	0.25	0.5	1	2	4	8	16	32	64	128	MIC_50_	MIC_90_
Erythromycin	27 *	13	33	23	16		1		4		1	4
Tylosin	5 *	4	3	8	20	36	22	17	1	1	8	32
Tilmicosin	26 *	12	11	31	15	14	3	3	1	1	2	8
Tulathromycin	16 *	18	26	27	22	5	1		0	2 *	1	4
Gamithromycin	85 *	13	11	4	2				2		<0.25	1

* Number of isolates with MICs equal to or higher than the test range. MIC, minimum inhibitory concentration.

**Table 2:** MIC values of five erythromycin-resistant isolates and associated resistance mechanisms.

Strains	MIC Values (μg/mL)					Resistance Gene/Mutation	Location
	ERY	TYL	TIL	TUL	GAM		
20	16	32	32	8	4	None	None
H62	64	128	128	>128	64	A2059G	23S rRNA
59	64	16	64	16	2	*erm*(T)	Plasmid
H44	64	64	32	>128	64	*erm*(T)	Chromosome
H68tg	64	32	32	8	2	*erm*(T)	Chromosome

ERY, erythromycin; TYL, tylosin; TIL, tilmicosin; TUL, tulathromycin; GAM, gamithromycin; MIC, minimum inhibitory concentration.

## Data Availability

The assembled genome sequences were deposited in NCBI under BioProject accession numbers SAMN44656869, SAMN44656860, SAMN44656893, SAMN44656902, and SAMN44731223.
